# Quantification and Statistical Analysis Methods for Vessel Wall Components from Stained Images with Masson's Trichrome

**DOI:** 10.1371/journal.pone.0146954

**Published:** 2016-01-13

**Authors:** Pablo Hernández-Morera, Irene Castaño-González, Carlos M. Travieso-González, Blanca Mompeó-Corredera, Francisco Ortega-Santana

**Affiliations:** 1 IUMA Information and Communication Systems, University of Las Palmas de Gran Canaria, Campus Universitario de Tafira, Las Palmas de Gran Canaria, Spain; 2 Department of Dermatology, Doctor Negrin University Hospital of Gran Canaria, Las Palmas de Gran Canaria, Spain; 3 Institute for Technological Development and Innovation in Communications (IDeTIC), University of Las Palmas de Gran Canaria, Campus Universitario de Tafira, Las Palmas de Gran Canaria, Spain; 4 Department of Morphology, University of Las Palmas de Gran Canaria, Campus de San Cristobal, Las Palmas de Gran Canaria, Spain; 5 CliniVar, Clínica de Varices, Las Palmas de Gran Canaria, Spain; 6 Department of Telematic Engineering, University of Las Palmas de Gran Canaria, Campus Universitario de Tafira, Las Palmas de Gran Canaria, Spain; University of Palermo, ITALY

## Abstract

**Purpose:**

To develop a digital image processing method to quantify structural components (smooth muscle fibers and extracellular matrix) in the vessel wall stained with Masson’s trichrome, and a statistical method suitable for small sample sizes to analyze the results previously obtained.

**Methods:**

The quantification method comprises two stages. The pre-processing stage improves tissue image appearance and the vessel wall area is delimited. In the feature extraction stage, the vessel wall components are segmented by grouping pixels with a similar color. The area of each component is calculated by normalizing the number of pixels of each group by the vessel wall area. Statistical analyses are implemented by permutation tests, based on resampling without replacement from the set of the observed data to obtain a sampling distribution of an estimator. The implementation can be parallelized on a multicore machine to reduce execution time.

**Results:**

The methods have been tested on 48 vessel wall samples of the internal saphenous vein stained with Masson’s trichrome. The results show that the segmented areas are consistent with the perception of a team of doctors and demonstrate good correlation between the expert judgments and the measured parameters for evaluating vessel wall changes.

**Conclusion:**

The proposed methodology offers a powerful tool to quantify some components of the vessel wall. It is more objective, sensitive and accurate than the biochemical and qualitative methods traditionally used. The permutation tests are suitable statistical techniques to analyze the numerical measurements obtained when the underlying assumptions of the other statistical techniques are not met.

## Introduction

The conditions of blood flow in the arterial and venous systems determine some of its structural features. Its basic composition follows a pattern in concentric layers, from the inside outward, called intima, media and adventitia. The arterial changes are less frequent, but more dangerous to the integrity of patients because they can lead to the production of aneurysms, with the danger of breaking and bleeding, or ischemic processes in any of the body organs, causing heart attacks or irreversible necrosis on many occasions.

Venous system disturbances are typically much less hazardous but, on the contrary, occur frequently in some cases. Such is the case of venous insufficiency, often manifest as lower limb varicose veins, one of the most common disorders in Western countries and the most common peripheral blood vessel complaint. Varicose veins affect up to 40% in men and 32% in women [1,2], since early clinical stages may cause deterioration in patients’ quality of life [3].

Veins are equipped with one-way valves, which prevent blood flowing backward (reflux). When the valves fail, blood accumulates in the veins and, over time, the veins become tortuous, creating the visible bulging known as varicose veins. The valve failure condition is known as valvular insufficiency.

At this time the cause of venous insufficiency is not known. Despite valvular insufficiency always exists, it is not clear whether the incompetence triggers the process or if certain changes in the structural components of the vessel wall determine the loss of tone and the subsequent venous dilatation [4–6].

**Fig 1:**
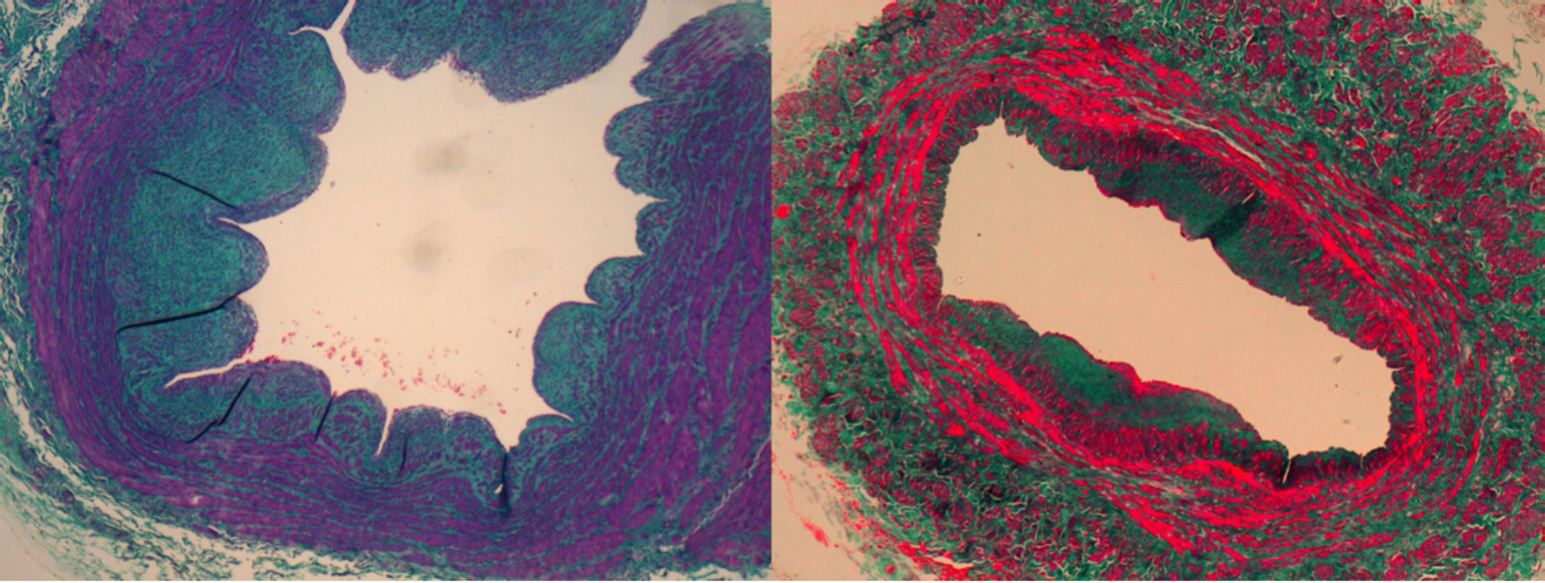
Color variation in two samples stained with Masson’s trichrome.

Histological staining vascular studies reveal the underlying tissue structures. The Masson’s trichrome stain is used to differentiate extracellular matrix (ECM) and smooth muscle fiber (SMF) in a tissue. Fig 1 shows two stained sections of long saphenous vein, with a central blood-containing space called lumen surrounded by the vessel wall with two distinct colors: a reddish tone and a bluish or greenish tint, depending on which technique variant was used. The structures stained in red correspond to SMF while dyed bluish or greenish hues correspond to the ECM in which many elements such as collagen and elastin are arranged. In cases of venous insufficiency, both the distribution and the relationships between SMF and ECM are impaired, thus reducing the tone of the vein wall, the progressive dilatation of the vein and, finally, the appearance of varicose veins.

The observation and measurement methods used on histological samples in published studies vary widely. While some studies use only exploratory and qualitative observation by optical or electron microscope, merely seeking to understand the phenomenon [4,7–9], others complement microscopic observation with the quantification of some parameters. Traditionally, vessel wall quantification has been performed using both biochemical [10–13] and stereological methods [14]. Morphological changes were described under the subjective interpretation of the observers of the samples. The lack of unified criteria prevents both comparing the results of different studies as well as the possibility of objective correlations between the findings and the different epidemiological factors related to venous disease.

It should be noted that the results and conclusions of the studies conducted are mismatched, which is attributable to the different methodologies used, typical of the era in which they were conducted. For example, the use of certain non-selective markers in biochemical analysis such as hydroxyproline results in inaccurate measurements [15], as well as the practice of expressing the content of these markers in relation to weight dry tissue. The latter was very common in the first published studies but could be misleading if the absolute mass of the vein wall is modified or altered, a phenomenon known as "reference trap" [14]. Another source of disparity is the subjectivity of qualitative methods used for describing some features, and therefore susceptible to variations on the observations of an investigator at the time, and between observations of different researchers, noting that a trained eye cannot provide an accurate measurement due to the limitations of human visual perception [16,17].

Significant progress has been made in the field of image analysis, increasing its capacity and precision, and the recovery of invisible information to the human eye. This has resulted in a wide range of analysis images applications aimed at solving various tasks in tissue diagnosis and research. The spectrum of these tasks is wide: from simple morphometry of cells and tissue structures and sub-cellular molecular studies to multispectral image analysis [16].

Digital image analysis allows more objective, sensitive and accurate quantitative assessments than visual qualitative methods. All of these techniques are applied to images to improve their quality, interpretation or provide tools to extract information from them [18]. The methods of image segmentation provide an initial approximation of the structure of interest based on morphological characteristics such as size, shape, color, etc., enabling the discrimination of smaller areas visually imperceptible, and later quantifying of the elements [17].

The image processing techniques applied in each step depend on the medical specialty in which they apply due to the different image characteristics in each field of study. For example, the histology images are in color with many objects of interest identified, while radiologic images display grey shades with only one or two objects in them. In addition, histological images may be affected by various factors that hinder analysis, such as overlying tissues, folds, variations in color and brightness, blur areas, etc.

A growing number of studies in many areas of biology and medicine apply digital image analysis. However, to the best of our knowledge, its application in the analysis of the insufficient vein wall is very low and the methodology has not been sufficiently clarified. Recent studies utilize these techniques, i.e. Elsharawy et al. [5] use an image analyzer called *Super-Eye* to measure layer thicknesses and area percentages of some vessel wall components, or Regadera et al. [19] who perform morphometric quantification of actin and elastic fibers using the *ImageJ* program. However none of these publications describe the digital image processing methods used.

On the other hand, if we analyze the sample sizes of these studies published in biomedical journals, we note that the number of subjects is variable and small, and generally broken down into subgroups of a particular independent variable of interest, thus further reducing the subgroup size. The small sample sizes reduce statistical power and increase the chance of making a type II error, that is, incorrectly concluding that the null hypothesis is true.

The parametric statistical methods impose certain requirements on data supporting its validity as the normal distribution of data, homogeneity of variances, etc. In above conditions with small sample sizes, we can accept the null hypothesis when testing the normality assumption, even when the data do not represent graphically such behavior. Moreover, nonparametric methods are distribution free (they do not impose the condition of normality) but they are not assumption free. Although they do not require a particular distribution, some assumptions must be met as symmetric distributions or similar shapes and variances among the groups to be compared [20]. We propose the use of resampling techniques, which are more robust [21]. Resampling techniques do not require the normal distribution, samples with large sizes, the homogeneity of variances or the similarity of distributions to compare [22].

The main contributions and innovations of this work are:

1. A database with 48 images of segments of great saphenous vein from patients with chronic venous disease undergoing valve reflux, without valvular reflux and healthy segments. As far as we know, there is no publicly available database.
2. Design and implementation of a two-stage method: pre-processing and feature extraction that allows less subjective quantification of different structural components of the vein wall (smooth muscle fiber and extracellular matrix) using techniques of digital image processing.
3. An inferential statistical method based on resampling techniques for the analysis of the numerical data obtained from the vein wall, applicable to small sample sizes, where the requirements of traditional statistical methods cannot be properly verified. The implementation on a multicore architecture is proposed to reduce the execution time.

The paper is organized as follows: The next two sections introduce the pre-processing stage that results in the delimitation of the study region and the feature extraction stage where the components of the vessel wall are segmented and quantified. The next section presents the image database where the previously described method was applied. The numerical results are presented and the different types of veins are statistically compared. In a final discussion, this paper explains the contribution of the proposed method in the field of study. Finally, the Conclusion section contains the conclusions and future work.

## Pre-Processing Stage

**Fig 2:**
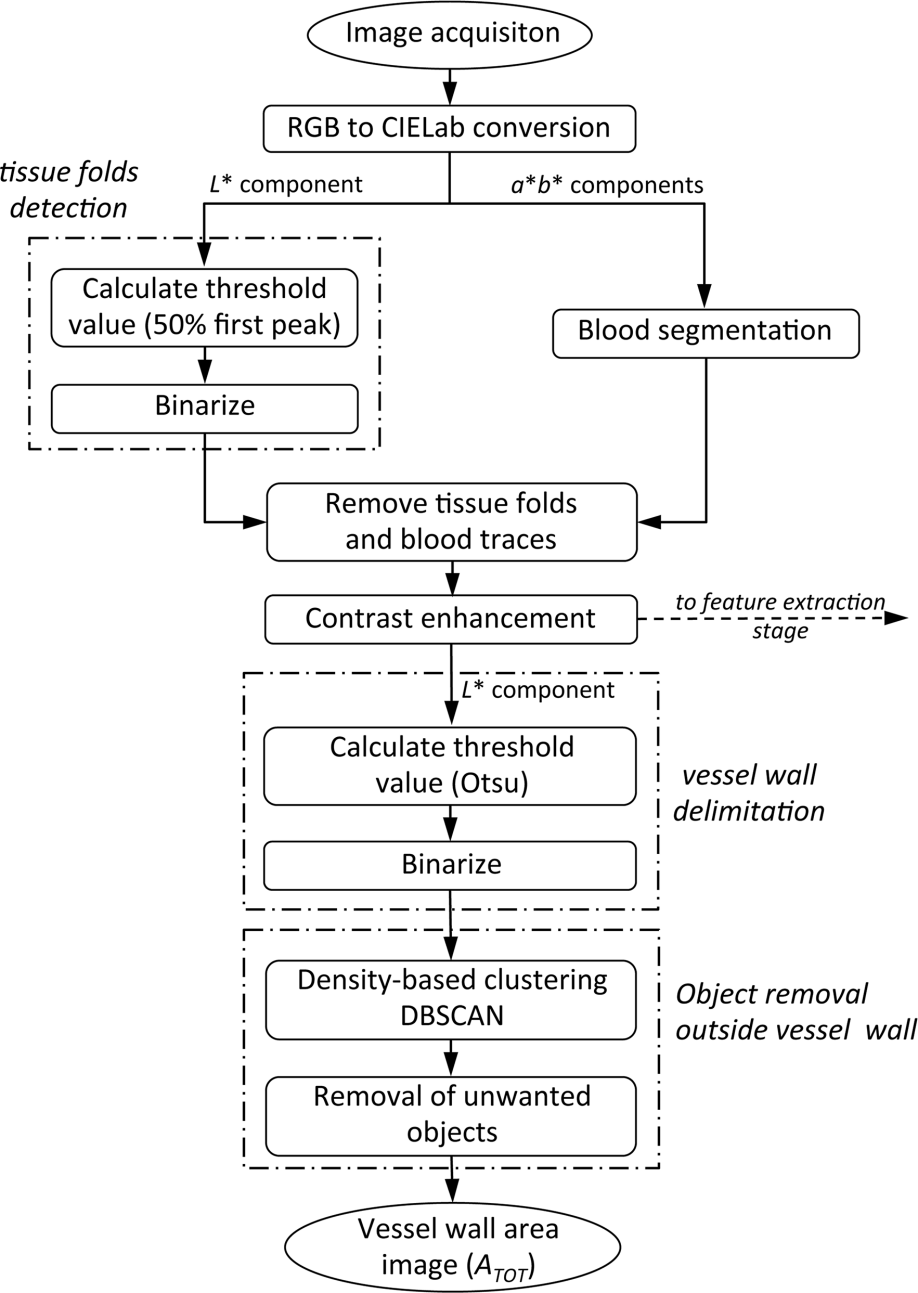
Flow chart of image pre-processing stage.

The goal of the pre-processing stage is to improve the appearance of the tissue image to clean the image and facilitate the delimitation of the study region. This stage focuses on color space conversion, enhancement contrast and area extraction. The result is a picture in black and white, with the vessel wall as a white object over a black background, which can be used to calculate the vessel wall area in pixels and as a mask in the next stage. Flow chart of this stage is given in Fig 2.

By its correlation with human visual perception the standard CIELab was chosen to characterize color channels, so that the images were converted from RGB to L*a*b* color mode, where color is described by three values: the lightness (L*), a value in the spectrum of colors ranging from green to red (a*) and a value in the colors ranging from blue to yellow (b*). As the grayscale representation of the image we will use the lightness component, which is computed by a nonlinear transformation of the RGB channels.

Initially two image segmentation task are performed in order to identify tissue folds and spots on the vessel wall, and moreover blood residues in the image which could cause some bias in the measurement of the SMF component due to the similarity of their colors.

**Fig 3:**
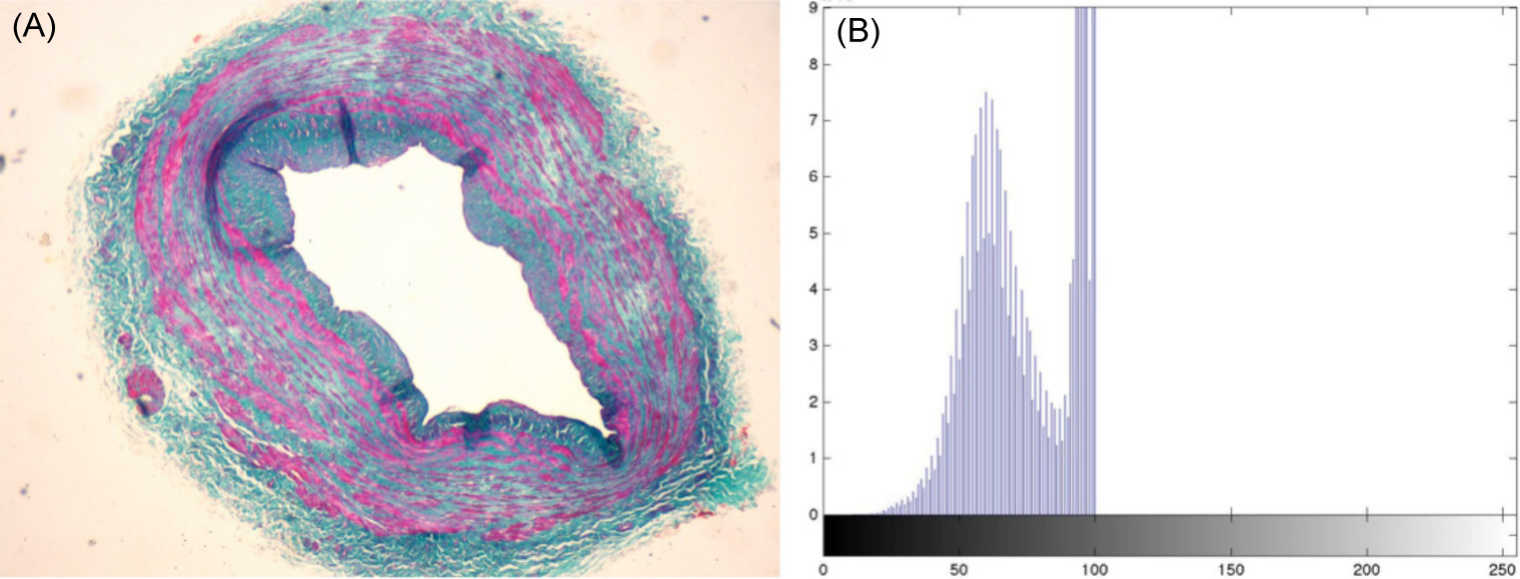
CIELab image. (A) Vessel wall image; (B) Histogram of the lightness component.

Tissue folds are segmented from the image, as they may lead to misinterpretations since its color does not indicate a clear distinction between the existing components, ECM or SMF. The tissue folds appear in the grayscale image as dark areas, so binarizing the grayscale image with a low threshold value identifies them. This threshold value is calculated as the halfway point between 0 and the location of the first predominant peak in the lightness component histogram. Fig 3B shows the histogram of the lightness component of the vessel wall image in Fig 3A, where the peak, on the left side of the histogram, corresponds to the vein wall followed by two peaks corresponding to the outside wall and the lumen.

Blood traces are segmented by color threshold detecting image objects that fall within a specified color range known as color mask. The color mask is a range centered in a color selected after analyzing the blood traces of a subset of our samples. The width range allows some degree of variability since certain blood color variation exists among the different samples.

**Fig 4:**
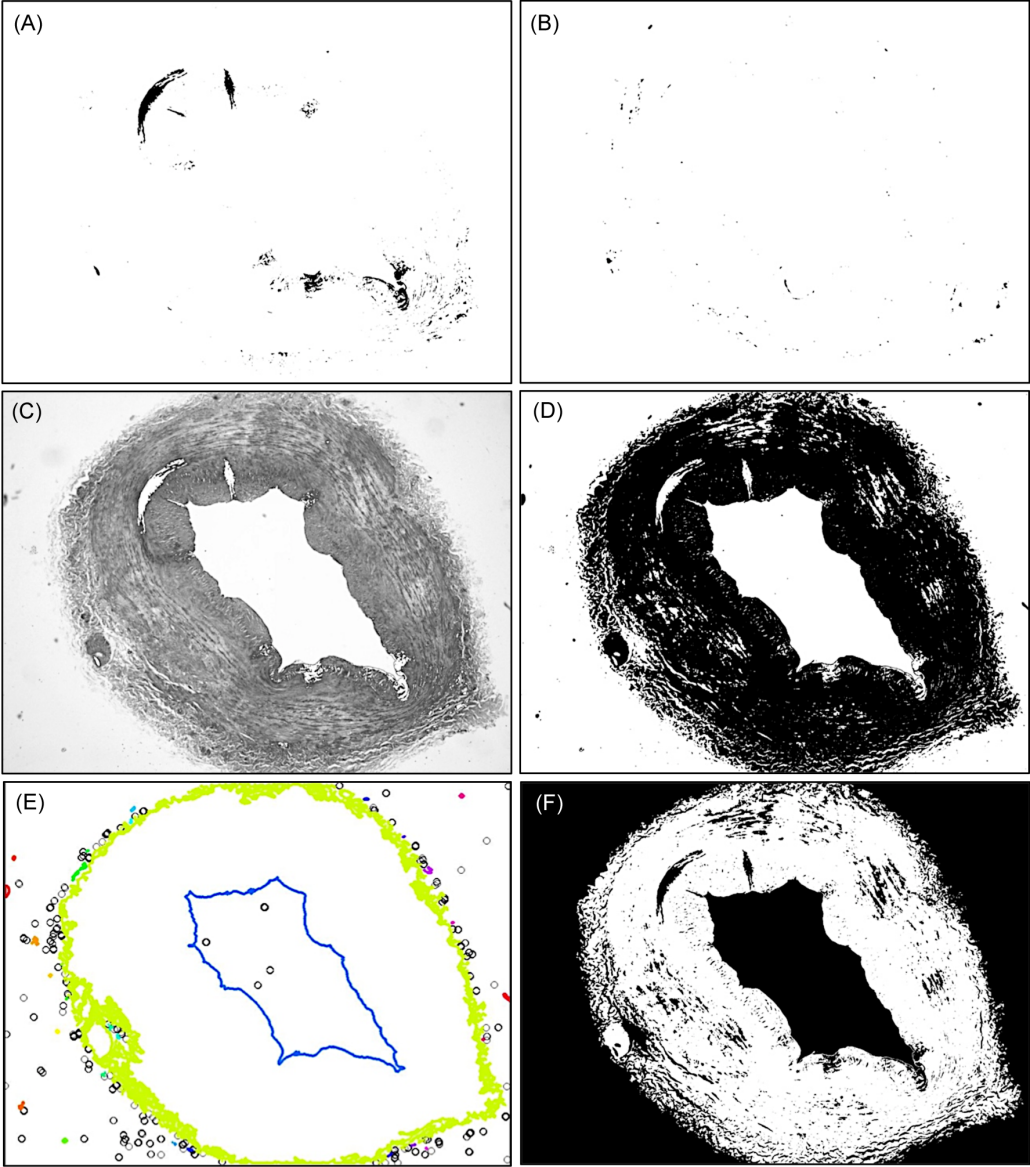
Pre-processing stage. (A) Tissue folds detected; (B) Blood residues detected; (C) Lightness component of the contrast-enhanced image after removing defects; (D) Binary image thresholded by Otsu’s method; (E) DBSCAN algorithm output; (F) Vessel wall area image.

Fig 4A and Fig 4B show the segmented tissue folds and blood traces of the vessel wall image in Fig 3A, where segmented regions are represented as black objects.

The segmented tissue folds of the vessel wall and the segmented blood traces of the image are used as binary mask, and they are multiplied pixel-wise by the L* component of the original image to remove all this image information that is of no interest. Each pixel in the lightness component of the image is multiplied by the corresponding mask pixel (value 0 or 1).

Contrast enhancement of color image was done by stretching the lightness component. Manipulating luminosity affects pixel intensity, while preserving the original colors. This transformation remaps each lightness level pixel *L* in the range [*L*_*min*_, *L*_*max*_] to the entire intensity range [0, 100] by the following expression:

$$round\left[100*\frac{\left(L-L_{min}\right)}{\left(L_{max}-L_{min}\right)}\right]$$ (1)

The resulting image will be one of the inputs of the feature extraction stage, and from here we will continue the process described below only with the lightness component of the contrast-enhanced image. Fig 4C shows the lightness component of the contrast-enhanced image after removing of the tissue folds and blood residues.

At this step of the process, the grayscale image of the lightness component is binarized by a threshold value *T* in the range [0,1] resulting in a binary image. The choice of the *T* value is done to show the vessel wall as a black object over a white background. The ideal threshold value depends on aspects such as the lighting conditions in which the vessel wall's picture was taken, the existence of shadows, particles of dirt, etc., so that its value is determined individually for each image. The threshold value is calculated by applying Otsu’s method on the processed image. Otsu’s method chooses the optimal threshold by maximizing the between-class variance (pixels in foreground or background) or by minimizing within-class variance with an exhaustive search through all the possible threshold values and measuring the spread for the pixel levels at each class [23]. Fig 4D shows the binarized image of Fig 4C with the threshold value calculated by Otsu’s method.

Finally, small objects of dust and dirt present in the binarized image outside the vessel wall and inside the lumen are identified and removed by a density-based clustering algorithm known as DBSCAN (Density-Based Spatial Clustering of Applications with Noise) [24].

Density-based clustering has its basis on the concept of neighborhood. The neighborhood of a data point *dp* is defined as the set of points that are contained in a circle of radius predefined *R*, centered at *dp*. The concept of density for a neighborhood can be seen as the number of data points contained within the neighborhood.

The DBSCAN algorithm is applied to a binary image considering foreground pixels as data points. Given an image, DBSCAN groups foreground pixels that are closely together, and marking as *noise* those foreground pixels that lie alone in low-density regions (whose nearest neighbors are too far away). Basically the algorithm finds neighbors of foreground pixels within a circle of radius *R*, and adds them into the same cluster if the number of neighbors in the circle contains at least a predefined minimum number of foreground pixels *MinPxs*.

The DBSCAN algorithm starts by randomly selecting a foreground pixel *px* of the image. If the neighborhood of *px* does not contain *MinPxs* foreground pixels it will be marked as noise and not assigned to any cluster, otherwise it will be included in the current cluster, and this process will continue with all foreground pixels that are directly-reachable from *px* until the density-connected cluster is completely found. Then, a new unclassified foreground pixel will be selected and processed, leading to the discovery of a further cluster or noise. The algorithm ends when all foreground pixels have been properly classified either assigned to a cluster or designated as noise.

Fig 4E shows the output of DBSCAN where clusters are visualized by different colors and noise pixels are represented by black circles. In the figure, the larger clusters correspond to the outer vessel wall (in light green color) and to the lumen (in blue color). These are the clusters we want to keep, while the rest of clusters or noise pixels would correspond to unwanted objects that will be eliminated from the image. Once these unwanted objects have been deleted the resulting binary image is shown in Fig 4F, which will be used as a mask to define the portion of the original image corresponding to the vessel wall.

## Feature Extraction Stage

**Fig 5:**
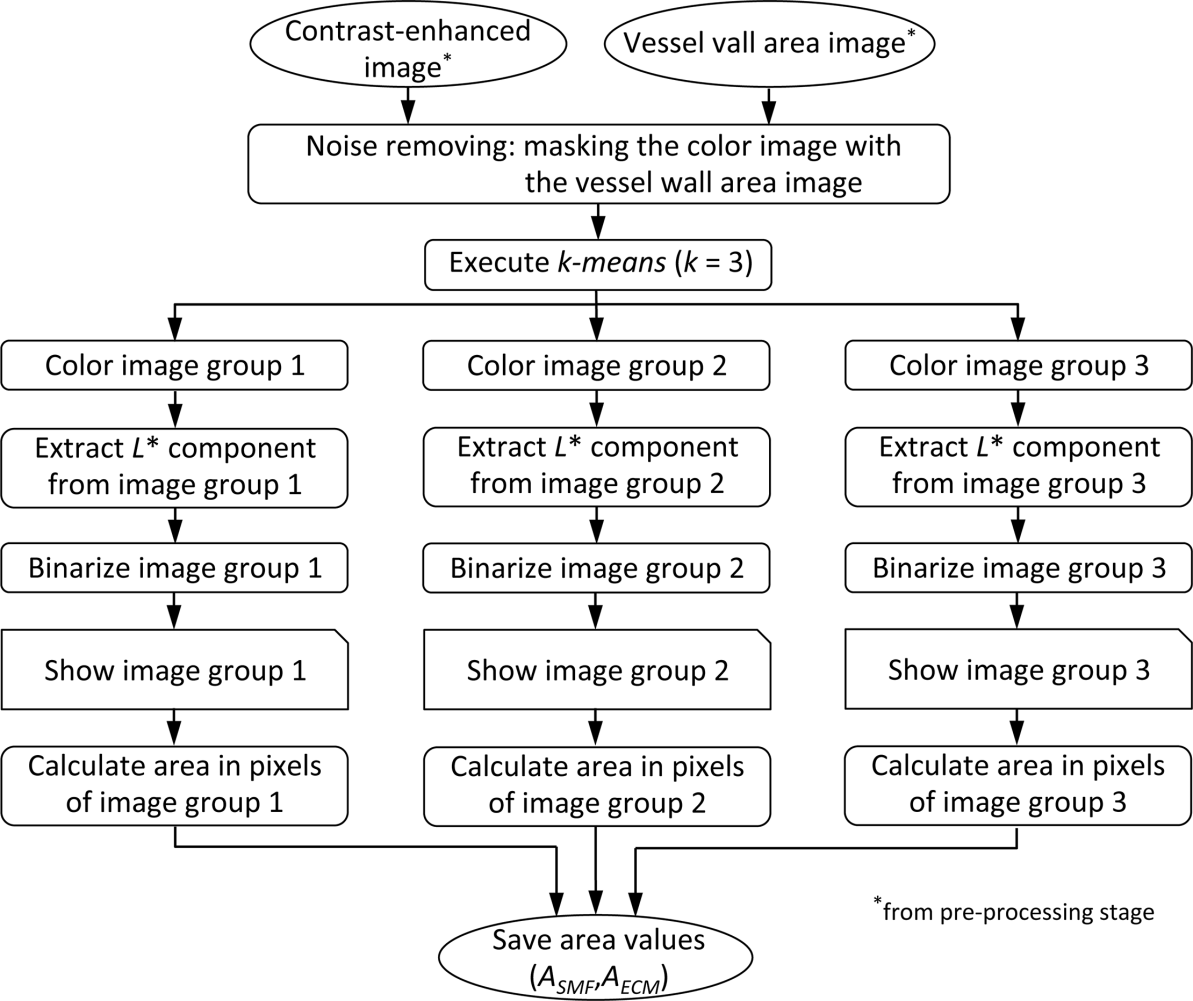
Flow chart of image feature extraction stage.

The feature extraction stage consists of an image content analysis based on the pixel color. The vessel wall image is clustered automatically in a predefined number of color-based classes. The results are several binary images, one per cluster, with the segmented vessel wall component, which allows us to calculate its extension. The flow chart of this stage is given in Fig 5.

**Fig 6:**
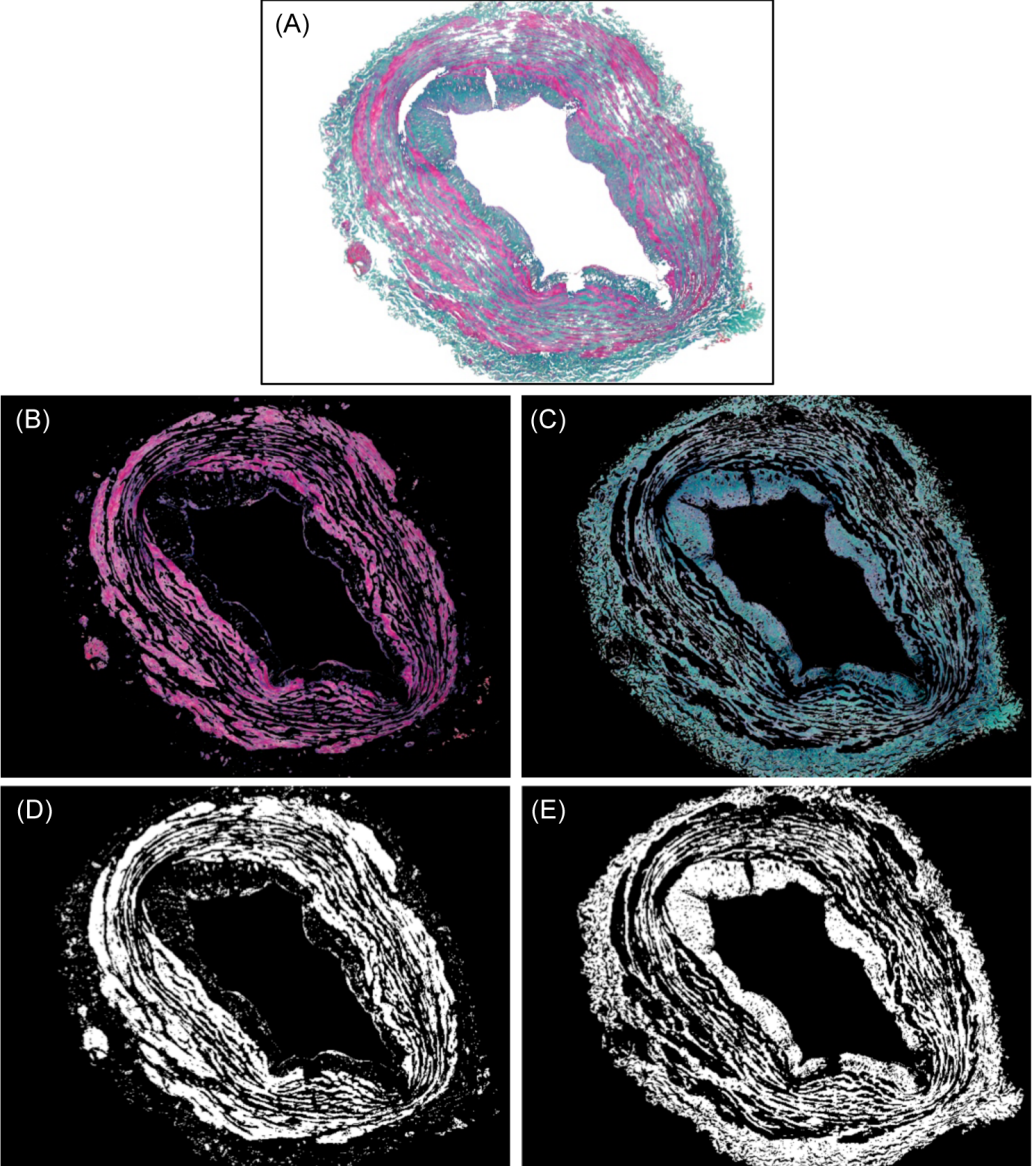
Process of the *k-means* algorithm. (A) Input image; (B) SMF component detected; (C) ECM component detected; (D) SMF area; (E) ECM area.

This stage starts masking the contrasted-enhanced image by the vessel wall area image obtained in the pre-processing stage. In this way, all objects not included in the vessel wall are removed from the color image and the background color is established as white. Fig 6A shows the resulting image with the vessel wall components the same two colors as the original and the background in white color, after masking the image of Fig 3A by the binary image in Fig 4F.

Then, the SMF and ECM components of the vessel wall will be grouped by a clustering algorithm based on the similarity between pixel colors. The clustering algorithm used is known as *k-means* [25]. This automatic method does not need prior calibration of color and is not limited to 2 or 3 colors. In addition the results are not affected by possible variations in color between different samples of the same staining as detected in our samples (see Fig 1).

The *k-means* clustering algorithm starts with a single group that includes all pixels and which is subdivided into as many groups as you like, so it is previously necessary to specify the number of desired groups (parameter *k*).

Initially the algorithm randomly chooses *k* pixels called centroids, each of which represents each of the *k* groups. Next, the remaining pixels are assigned to the group that has more similarity by comparing the pixel with each of the *k* centroids. The result is a first distribution of the whole set of pixels in *k* groups. In clustering algorithms based on color, the similarity between two pixels *px*_1_ and *px*_2_ is measured by calculating the Euclidean distance between the color components of each pixel, and in the case of encoding L*a*b* is:

$$distance({px}_{1},{px}_{2})=\sqrt{{\left({L*}_{2}-{L*}_{1}\right)}^{2}+{\left({a*}_{2}-{a*}_{1}\right)}^{2}+{\left({b*}_{2}-{b*}_{1}\right)}^{2}}$$ (2)

so that the smaller the distance, the greater the similarity between pixels.

Following this, the *k-means* algorithm iterates to refine the content of the *k* groups. In each iteration, the centroid of each group is re-calculated as the average value of the colors of the pixels that compose it. These new values of centroids are used to reallocate pixels to each of the *k* groups. This process is done iteratively until a stopping criterion is reached, which is based on a cost function *J* defined as:

$$J=\sum_{j=1}^{k}\sum_{i=1}^{n_{j}}distance({px}_{i}^{j},{centroid}_{j})$$ (3)

where *n*_*j*_ is the number of pixels in group *j*, ${px}_{i}^{j}$ is the *i*-th pixel of the group *j*, and *centroid*_*j*_ is the centroid of the group *j*. In such a way that the process tries to minimize the cost function *J*, i.e. until the composition of the groups does not change or changes very little between adjacent iterations (lower than a pre-established threshold).

The cost function *J* is a non-convex function, so the minimum found may not be a global minimum, meaning that the result of *k-means* algorithm depends on the initial value assigned to the centroids, so it is a common practice perform multiple executions using random initialization for the centroids and select the clustering with lowest cost [25].

So, the number of groups *k* for the *k-means* algorithm was set at 3 with the aim of grouping the pixels corresponding to SMF, the pixels corresponding to ECM and the background image. The choice of the initial value of the centroids was random. The execution of the algorithm returns the group (value between 1 and *k*) to which each of the pixels in the original image belongs. Each of the three groups of pixels is moved to a different image. Fig 6B and 6C show the output images of the groups corresponding to the SMF and ECM component respectively. The lightness component of these images is binarized to get the binary images shown in Fig 6D and 6E. The third group of pixels corresponding to the background image is discarded.

In a binary image, the white color is coded with the value 1 and the black color is coded with 0. So the area, in pixels, identified by the white color can be calculated as the sum of all the pixels of the image. Thus the area in pixels of any component would be computed by the following expression on each binary image resulting from the *k-means* algorithm:

$$area(pixels)=\sum_{row}\sum_{column}pixel\_value(row,column)$$ (4)

## Experimental Methodology

This section describes the application of the pre-processing and feature extraction stages on a set of vessel wall images in order to characterize each type of vein. We then carry out the statistical analysis of the features extracted from the different vein types.

Firstly, we present the images database and a summary of the results obtained from these images after processing them. Then, a comparison of the numerical results is performed, where the use of resampling techniques is proposed because, when working with small sample sizes, the compliance with the requirements of traditional statistical methods cannot be properly verified.

### Image database

The database used in this work contains 48 histological sections of internal saphenous veins stained with Masson’s trichrome. They have been selected from the database of internal saphenous vein samples generated by Dr. Ortega-Santana, Dra. Mompeó-Corredera and Dra. Castaño-González. The sample selection was carried out by our medical team in accordance with the selection criteria described below for control samples and patients with venous insufficiency. Furthermore, we discarded those samples with non-assessable defective staining or with structural damage of the venous wall.

Given that the work focuses on a method of objectifying whether the structural composition of a tissue, in this case internal saphenous veins, is different when it is under different hemodynamic conditions, samples with different hemodynamic conditions were selected:

1. Control samples: These come from subjects undergoing judicial autopsy (Forensic Anatomical Institute in Las Palmas de Gran Canaria). In such cases, after checking through the medical history and physical inspection that the body showed no signs of venous insufficiency, during the act of the autopsy segments of internal saphenous veins were removed and immediately processed for histological studies.
2. Samples belonging to patients with venous insufficiency: Samples classified as "competent" and "incompetent" were obtained from patients diagnosed with venous insufficiency of the great saphenous vein. The procedures performed were as follows: the diagnosis was made by venous Doppler ultrasound, which allows anatomically mapping the extent and degree of venous insufficiency. After it the surgical procedure was explained to the patient in order to obtain their signed consent. During surgery and with the help of Doppler ultrasound, venous segments previously marked as competent (normal venous flow) or incompetent (retrograde venous flow, reflux) were located and the tissue samples were taken and immediately processed for histological studies according to standard techniques.

**Fig 7:**
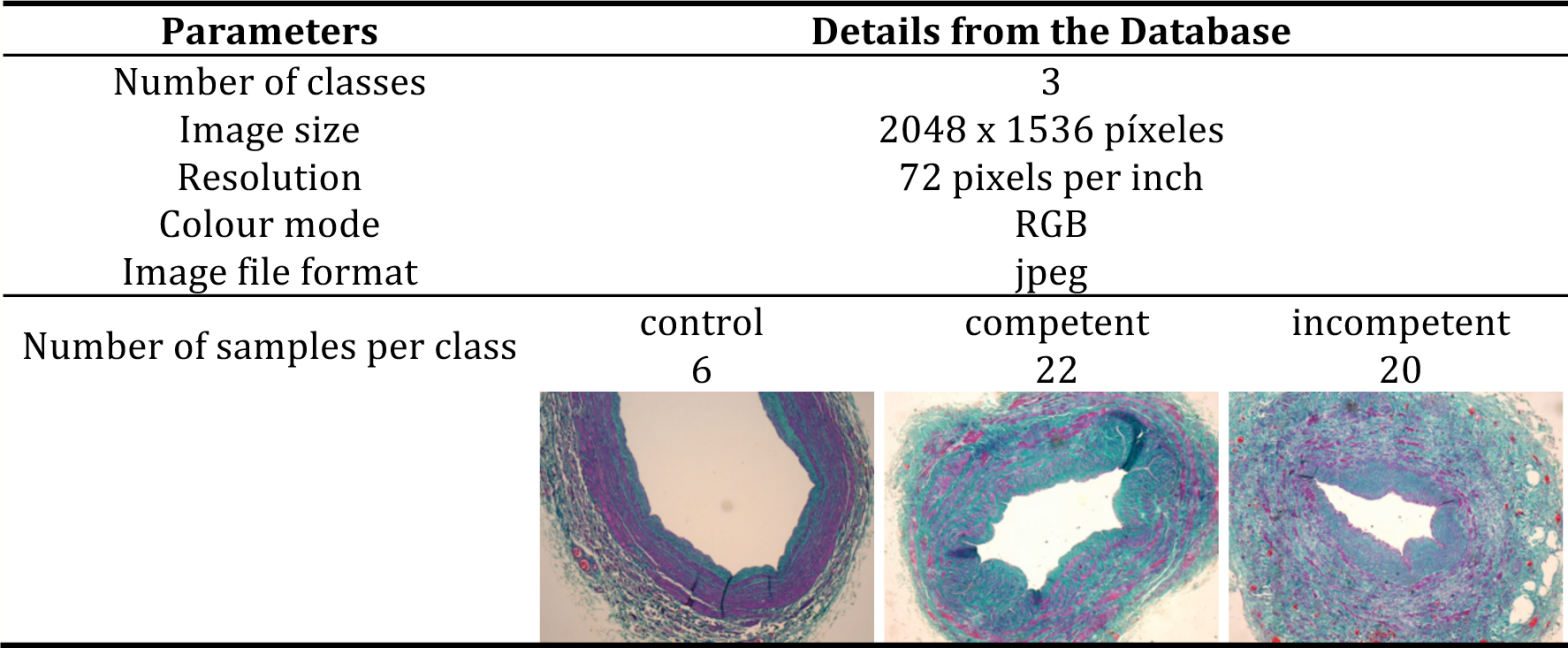
Characteristics of the database.

A microscope Leica Digital Microimaging Device 108 was used to acquire the images with a magnification 4x. A detailed description of this database can be found in Fig 7, which includes a thumbnail example per class.

### Ethics statement

All patients gave written consent for surgery so as to obtain samples with the aim to be used for scientific studies on venous insufficiency. Control samples were obtained and provided to our group by forensic doctors of Forensic Anatomical Institute in Las Palmas de Gran Canaria. The Ethics Committee on Human Research of University of Las Palmas de Gran Canaria approved the protocol for the collection and storage of samples and the protocol of this study.

### Experiments

Previously the doctors analyzed the database images by paying attention to the presence of smooth muscle fibers and extracellular matrix in the vessel wall. To validate the proposed methodology, the team of doctors performed a subjective rating in an independent way. The doctors analyzed the stained sample set and the presence of SMF and ECM in the vessel wall was evaluated by a grading with a score ranging from 0 (no presence) to 6. Their findings were not communicated to the team of engineers until completion of the study to check whether the results were similar.

The pre-processing and feature extraction approaches were implemented using MATLAB (version 7.12, R2011a—The MathWorks, Inc.) [26]. The pre-processing stage retrieves the image from the database, and generates two outputs: a contrast-enhanced color image and a binary image with the vessel wall area, which are stored in the local computer. Both images are input of the feature extraction stage. The feature extraction stage provides two measurements: *A*_*SMF*_, the area in pixels of the SMF component and *A*_*ECM*_, the area in pixels of the ECM component (see Fig 5).

Since the vein surface may be different in each sample, and in order to get comparable measurements between different samples it is necessary to normalize the area in pixels of each component by the area in pixels of the sample vessel wall. This value, *A*_*TOT*_, is calculated by the expression (4) on the resulting image of the pre-processing stage that was stored in the local computer (see Fig 2). So, the normalized measures of each sample will be obtained by the following ratios:

$${\bar{A}}_{SMF}=\frac{A_{SMF}}{A_{TOT}}\;{\bar{A}}_{ECM}=\frac{A_{ECM}}{A_{TOT}}$$ (5)

**Fig 8:**
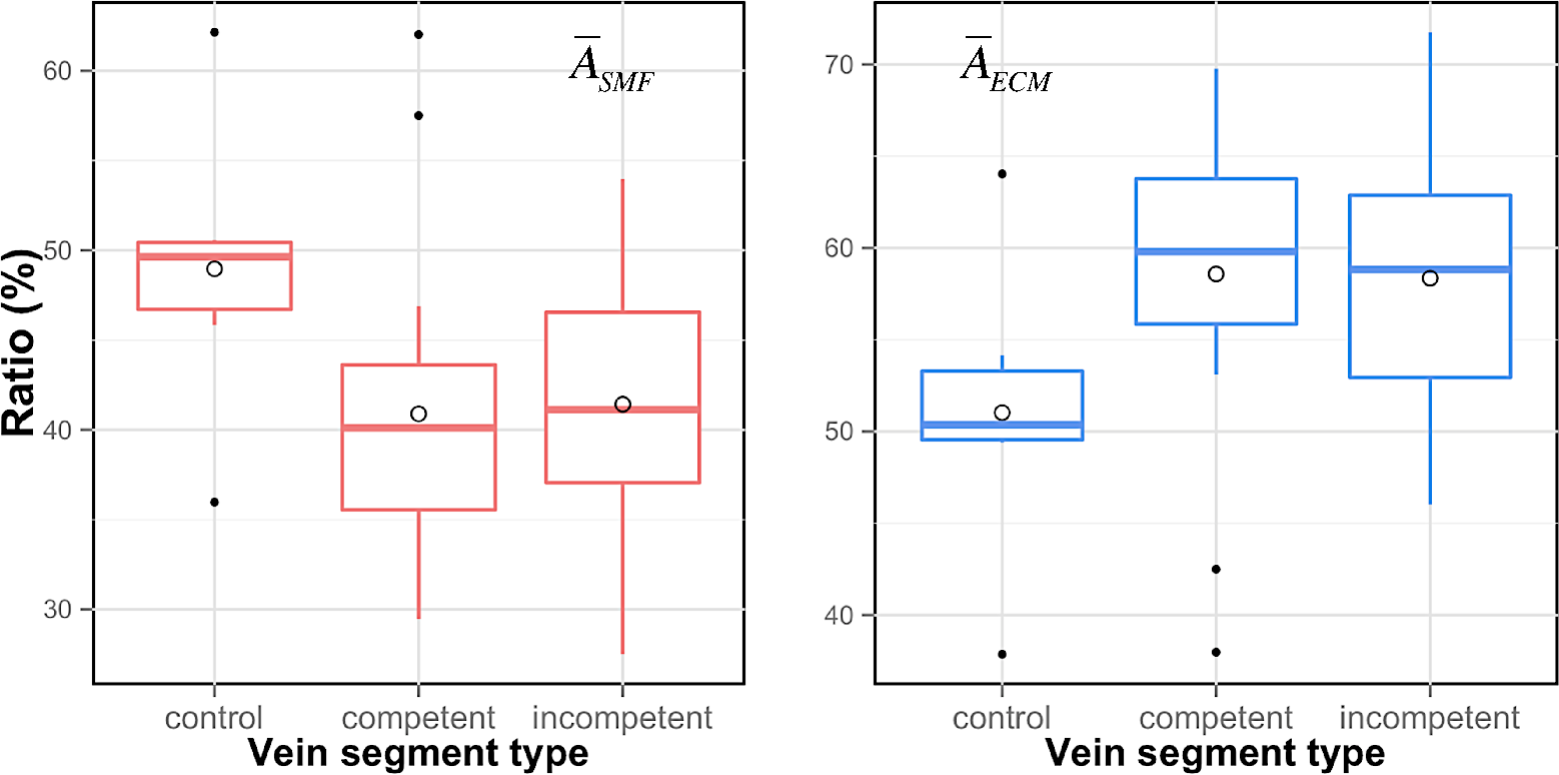
Normalized measures of different vein segment types.

Fig 8 shows the distribution of the ratios ${\bar{A}}_{SMF}$ and ${\bar{A}}_{ECM}$ for the 48 samples of our database, distinguishing among control, competent and incompetent veins. In each boxplot a circle indicates the average value.

### Statistical data analysis

Resampling techniques are an alternative to classical statistical tests, for making inferences based on the variability present in the available sample, rather than a particular assumption on the distribution of the population from which the sample was drawn. Among the major types of resampling, permutation tests were used for assessing the statistical significance of the differences observed among the groups. The permutation test provides an alternative approach that does not require any assumptions about normality, the shapes of the distributions, etc. [22].

Under the null hypothesis of no differences between groups, the observed groups can be merged into a single group. This single group is resampled without replacement to obtain samples of the same size as the original groups, and the statistic of interest is calculated such as the difference in means or medians or the *F* value, generating a sampling distribution of an estimator under the null hypothesis. Then we compare the observed statistic in the original groups to this empirical sampling distribution to determine how unlikely our observed statistic is if the null hypothesis is true (*p*-value).

The variability of the results is inversely proportional to the amount of resampling performed, requiring a large computational power and memory resources [27]. In the case of 2 groups of size *n*_1_ and *n*_2_, the number of all possible combinations of group membership is given by the following expression:

$$\frac{(n_{1}+n_{2})!}{n_{1}!∙n_{2}!}$$ (6)

When samples are of modest size, it is more practical to work with a limited number of resamplings. When working with a subset *m* of all possible combinations and in order to avoid that the *p*-value can be exactly zero, it must be calculated as:

$$p\_value=\frac{b+1}{m+1}$$ (7)

where *b* is the number of times yielding an empirical test statistic at least as extreme as that observed from the original data (one-tailed test) [28].

As the number of resamples increases the execution time of the permutation test is greater. Parallelizing code execution across multiple processors can reduce this time. Table 1 shows the execution time of a permutation test to analyze statistically the differences between competent and incompetent vein classes and the 95% confidence interval (CI) for the *p*-value with different number of resamplings. The group sizes are 22 and 20 elements (as indicated in Fig 7), so there are 5.138*10^11^ different combinations according to Eq (6). Permutation test were implemented by R software version 3.0.2 on a MacBook Pro with an Intel Core i5 2.6 GHz with 2 cores (4 virtual cores) and 8 Gb RAM.

**Table 1:** Execution time of permutation test (*n*_1_ = 22 and *n*_2_ = 20).

Number of resamplings	Execution time		*p*-value CI (95%)^*^
	Sequential	Parallel	
10,000	0.84 secs	1.82 secs	[0.8104376, 0.8255624]
100,000	7.69 secs	4.29 secs	[0.8191065, 0.8238535]
1,000,000	1 min 13.4 secs	32.69 secs	[0.8193892, 0.8208948]
10,000,000	12 min 17.6 secs	5 min 5.1 secs	[0.8195452, 0.8200216]
100,000,000	2 h 2 min 52.5 secs	50 min 39.6 secs	[0.8196250, 0.8197757]
1,000,000,000	20 h 35 min 9.7 secs	8 h 30 min 11.3 secs	[0.8196984, 0.8197460]

* CI on the assumption that *p*-value follows a binomial distribution

Statistical significance of the differences between the types of vein segments in Fig 8 was calculated by permutation tests. A useful guideline is to start with a small number of resamplings and increase the number of resamplings only if the *p*-value obtained is near the significance level of our hypothesis testing. The initial number of resamplings was set in 100,000 that imply that the maximum resolution for the *p*-value was 10^−5^ and the uncertainty near our significance level will be about 0.14%. The statistic of interest was the difference in the arithmetic means of both groups and the null hypothesis states that there is no difference between the two population means. S1 Appendix shows the R script to perform the parallel computation of the permutation test using the package *parallel* [29], which was introduced in R version 2.14.0 and integrates functionalities of several existing packages of parallel computation.

With a significance level of 0.05, the *p*-values from permutation test obtained for both vessel wall components show that there is no statistical difference between competent and incompetent veins groups, while the difference between control group and both groups of varicose veins is statistically significant. Table 2 shows the *p*-values.

**Table 2:** *p*-values from experiments.

*SMF*	Control	Competent	*ECM*	Control	Competent
Competent	0.03765962	—	Competent	0.0408496	—
Incompetent	0.03624964	0.8217618	Incompetent	0.0395446	0.9181308

Doctors reported that the structure of the vein wall of the control group was consistent with normal histological description whereas samples obtained from patients with venous insufficiency (both competent and incompetent groups) showed a clear change in the amount and the distribution of both the extracellular matrix and smooth muscle fibers (see images displayed in Fig 7). They concluded that there were differences between the control group and pathological samples but could not say whether there were differences on the ECM or SMF amount between competent and incompetent segments.

### Discussion

**Fig 9:**
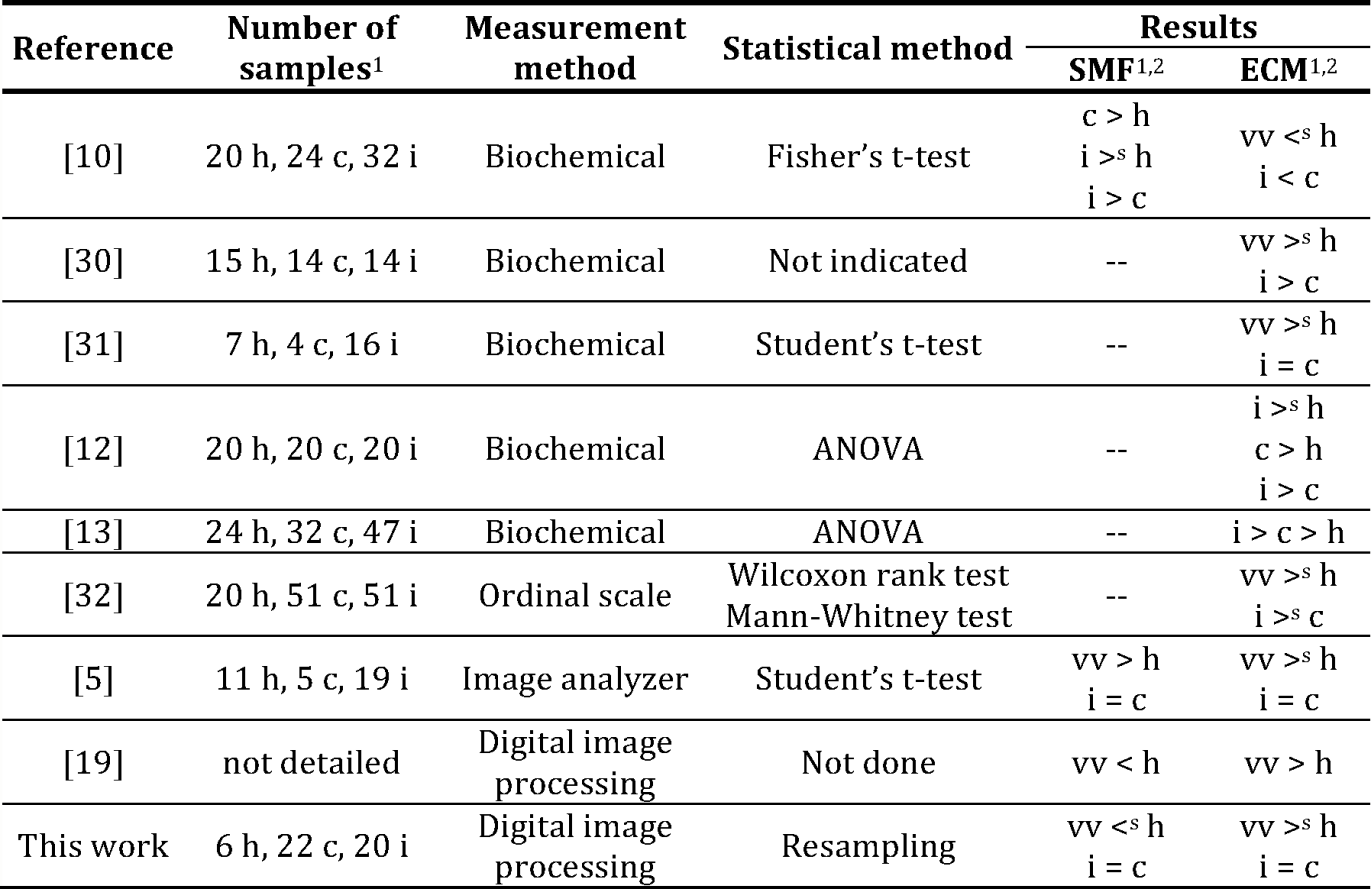
State-of-the-art in varicose vein studies. ^1^ h: healthy sample, c: competent sample, i: incompetent sample ^2^ vv: varicose samples (competent and incompetent). <^s^ or >^s^: the difference indicated is statistically significant (*α* = 0.05). < or >: the difference indicated is not statistically significant.

Numerous studies have compared vein segments at different stages of disease: healthy veins (controls), veins with absence of reflux (competent) and veins with presence of reflux (incompetent), in order to identify the distinctive features of each of them by different methods of observation and measurement. Fig 9 shows the main characteristics of the studies carried out by several authors [5,10,12,13,19,30–32], where the size of the sample, the quantification method, the statistical method and the results obtained are indicated.

The results of the studies listed in Fig 9 are not similar and sometimes are opposite such as the ones corresponding to ECM reported by Svejcar et al. [10] and Elsharawy et al. [5], where Svejcar et al. indicate that varicose samples, that is, competent and incompetent samples, contain less ECM than healthy samples and these differences were statistically significant and instead, the results of Elsharawy et al. show a significantly higher amount of EMC in varicose samples compared to healthy samples. Although most authors identify the contradictions reported in the literature, they do not indicate a clear cause, which could be due, among others factors, to the different quantification methodologies employed. The quantification methods of the vein wall components vary: biochemical methods, semi-quantitative methods using ordinal scales, etc., and, recently, digital image analysis. However in the publications where quantification is done by digital images analysis [5,19], the pre-processing and analysis methods of digital images are not detailed.

At the same time, the list of studies in Fig 9 shows that parametric and non-parametric statistical methods are used ignoring the analysis of requirements, and therefore without considering the suitability of the chosen method.

## Conclusions

The main contribution of this work is to develop a methodology for segmenting and quantifying some vessel wall components by processing and analysis of digital image. The proposed methodology provides more objective measures during the histological analysis than the biochemical and qualitative methods traditionally used, despite the variability due to the random nature of some of the clustering algorithms used since its magnitude is small. Their use is desirable in order to reduce subjective bias in the manual evaluation in intra- or inter-operator variation problems.

The sample size in most studies using human tissue samples is usually very small and the use of traditional statistical tests may lead to unreliable results. It is necessary, therefore, to use suitable techniques to these sizes that do not verify the underlying assumptions of the techniques traditionally used.

Experimental results showed good correlation between the measured parameters and the doctors’ initial scoring (the Spearman correlation coefficient was 0.76 and 0.74 for ECM and SMF components respectively). These results show the consistency between the quantification methodology proposed and the subjective observation of the doctors for the evaluation of pathological changes in vessel wall.

Direct observation through a microscope does not always allow us to establish clear differences between two or more sample groups. Hence the importance of the method proposed since, in addition to quantifying the amount of any tissue present in the specific area of study, enhances rigorous statistical analysis that can help to more accurately know how it changes the structure of the venous wall in the case of the venous insufficiency.

The previous research have used different methods for determining the component amounts in the varicose vein wall but they have led to contradictory results. Therefore we hope this methodology will help to achieve an agreement about the wall structural changes to clarify the cause and evolution of varicose veins.

In the future, we will incorporate more samples to the database and extend the methodology for measuring other vessel wall component, i.e. the elastic fibers. In addition, we plan to extend the methodology to immunochemistry quantification of some markers by color slicing.

## Supporting Information

S1 Appendix R script for permutation test (two-tailed test). (TXT)
